# Polymyxin B as inhibitor of LPS contamination of *Schistosoma mansoni *recombinant proteins in human cytokine analysis

**DOI:** 10.1186/1475-2859-6-1

**Published:** 2007-01-03

**Authors:** Luciana S Cardoso, Maria Ilma Araujo, Alfredo M Góes, Lucila G Pacífico, Ricardo R Oliveira, Sergio C Oliveira

**Affiliations:** 1 Serviço de Imunologia, Hospital Universitário Professor Edgard Santos, Universidade Federal da Bahia, Salvador-Bahia, Brazil; 2 Escola Bahiana de Medicina e Saúde Pública, Salvador-Bahia, Brazil; 3 Instituto de Investigação em Imunologia (iii)/Conselho Nacional de Desenvolvimento Científico e Tecnológico, São Paulo-SP, Brazil; 4 Departamento de Bioquímica e Imunologia, Instituto de Ciências Biológicas, Universidade Federal de Minas Gerais, Belo Horizonte-MG, Brazil

## Abstract

**Background:**

Recombinant proteins expressed in *Escherichia coli *vectors are generally contaminated with endotoxin. In this study, we evaluated the ability of Polymyxin B to neutralize the effect of LPS present as contaminant on *Schistosoma mansoni *recombinant proteins produced in *E. coli *in inducing TNF-α and IL-10. Peripheral blood mononuclear cells from individuals chronically infected with *S. mansoni *were stimulated *in vitro *with recombinant Sm22.6, Sm14 and P24 antigens (10 μg/mL) in the presence of Polymyxin B (10 μg/mL).

**Results:**

The levels of cytokines were measured using ELISA. There was greater than 90 % reduction (p < 0.05) in the levels of TNF-α and IL-10 when Polymyxin B was added to the cultures stimulated with LPS. In cultures stimulated with *S. mansoni *recombinant proteins in the presence of Polymyxin B, a reduction in the levels of TNF-α and IL-10 was also observed. However, the percentage of reduction was lower when compared to the cultures stimulated with LPS, probably because these proteins are able to induce the production of these cytokines by themselves.

**Conclusion:**

This study showed that Polymyxin B was able to neutralize the effect of endotoxin, as contaminant in *S. mansoni *recombinant antigens produced in *E. coli*, in inducing TNF-α and IL-10 production.

## Background

Recombinant proteins represent an important tool in the field of immunology research and have been used to understand pathogenesis and host susceptibility and may serve as components for vaccines. In general these proteins augment the specificity of diagnostic tests since they are specific peptides with known sequences.

The production of most recombinant proteins is achieved through cloning into *Escherichia coli *vectors containing cDNA coding for the desired protein. A common limitation to this process is the contamination of recombinant protein with endotoxin [1]. Endotoxins are a set of complex lipopolysaccharides (LPS) which are the integral component of gram-negative bacteria such as *E. coli*. The LPS is a component of the pathogen associated molecular pattern (PAMPs) which bind to multiple receptors, such as LPS binding protein (LBP), macrophage receptor (CD14) and Toll like receptor-4 (TLR-4) [2,3]. This results in activation of intracellular signalling pathways and production of pro-inflammatory cytokine such as TNF-α, IL-1 and IL-6 [4,5]. In patients infected with gram-negative bacteria, high levels of TNF-α lead to an endotoxemic syndrome or septic shock, which can result in intravascular disseminated coagulation, heart failure and death [6]. It has been described that at a low dose, LPS is able to induce both TNF-α and IL-10 production [7]. IL-10 is an anti-inflammatory cytokine able to down-modulate TNF-α production [8,9]. After the process of recombinant protein production in *E. coli*, the protein is purified using different techniques. For example, proteins associated with 6-His tag fusion at the N-terminus are purified using an HPLC Ni^2+ ^charged resin (GE Health care). Proteins are eluted by imidazol in the refolding buffer. This is a very common method to obtain purified proteins, but they are almost always contaminated with certain amount of LPS. For instance, the commercially available recombinant proteins rHsp70 and rHsp60 induce TNF-α production by macrophages due to LPS contamination [8,9].

There are some methods to clean proteins from LPS contamination with limited efficacy [1]. The natural peptide, Polymyxin B (PMB), is a potent antibiotic that binds to and neutralizes LPS. It is a decapeptide cyclic cationic antibiotic containing lipophilic and hydrophilic groupment (lipophobic) that binds to lipid A, the major component of the endotoxin. The plasmatic half-life of PMB is 6 hours, but it can be detected in blood 8 to 12 hours after use. Studies have demonstrated that PMB can protect animals from the toxic effects of endotoxin [10,11]. In this study, we evaluated the ability of Polymyxin B to neutralize the effect of LPS present as contaminant in *Schistosoma mansoni *recombinant proteins produced in *E. coli *in inducing TNF-α and IL-10 production in peripheral blood mononuclear cells (PBMC) from individuals infected with *S. mansoni*.

## Results

**Figure 1:**
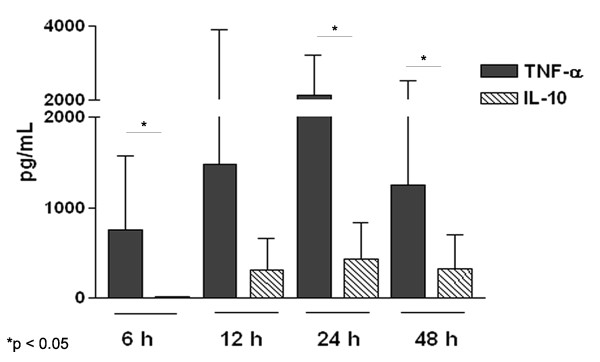
**LPS-induced production of TNF-α and IL-10 by PBMC**. This figure shows the kinetic of TNF-α and IL-10 production by PBMC of six individuals chronically infected with *S. mansoni *stimulated *in vitro *with LPS (0.14 ng/mL).

The kinetics of LPS-induced cytokine production was evaluated in PBMC cultures of individuals infected with *S. mansoni *incubated for 6, 12, 24 and 48 h. The concentration of LPS used in the cultures was similar to what was detected in the *S. mansoni *recombinant proteins used in this study (Table 1). We observed high levels of TNF-α in supernatants of LPS-stimulated cultures incubated for 6 h (758 ± 815 pg/mL), and the levels increased at 12 h and 24 h, 1482 ± 1489 and 2123 ± 1080 pg/mL, respectively (Figure 1). There was a decrease in TNF-α production after 48 h of culture, which represented a 40.4 % reduction. In contrast, IL-10 production began after 12 h of culture (314 ± 341 pg/mL), and reached a higher concentration at 24 h of culture (435 ± 395 pg/mL) (Figure 1).

**Table 1:** Levels of LPS present as contaminant in *S. mansoni *recombinant proteins P24, Sm14 and Sm22.6

*S. mansoni *Recombinant Protein (1 mg)	LPS	
	
	(EU/mL)	(ng/mL)
P24	1.35	0.135
Sm14	2.10	0.210
Sm22.6	1.32	0.132

**Figure 2:**
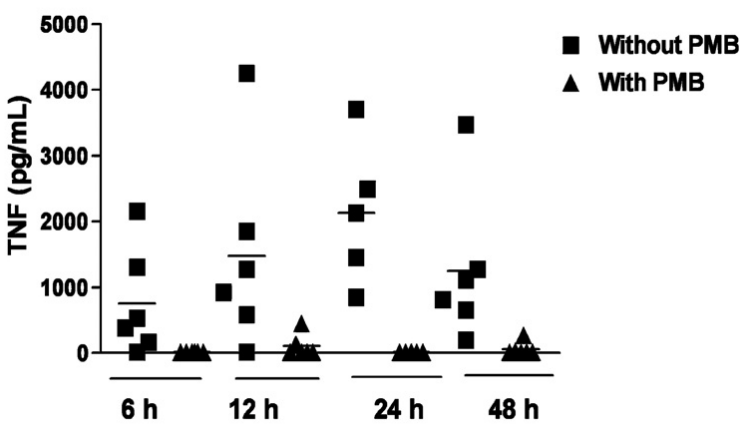
**Effect of Polymyxin B on LPS-induced cytokine production *in vitro***. This figure shows the effect of the addition of Polymyxin B to the PBMC culture of individuals chronically infected with *S. mansoni *(n = 06) on LPS-induced TNF-α. LPS was used in the concentration of 0.14 ng/mL and Polymyxin B at final concentration of 30 μg/mL of culture.

Since it is well known that LPS induces high levels of TNF-α [7], we evaluated the role of PMB blockage on the synthesis of this cytokine in cultures stimulated with LPS. The production of TNF-α in 6, 12, 24 and 48 h-cultures stimulated with LPS in the presence or absence of PMB is shown in Figure 2. Compared to cultures without PMB, there was a reduction in the levels of TNF-α by addition of this antibiotic to the cultures at all time-points evaluated. The mean levels of TNF-α decreased from 758 ± 815 pg/mL to 15.6 ± 9.0 pg/mL at 6 h-culture (97.9% of reduction, p = 0.03) and from 1481 ± 1489 pg/mL to 108 ± 175 pg/mL (92.7 % of reduction, p = 0.03), 2123 ± 1080 pg/mL to 15.6 ± 0 pg/mL (99.3 % of reduction, p = 0.06) and 1264 ± 1143 pg/mL to 60 ± 104 pg/mL (95.2 % of reduction, p = 0.03) at 12, 24 and 48 h-culture, respectively.

Polymyxin B was used at a final concentration of 30 and 60 μg/mL in cultures and similar levels of reduction in cytokine production were observed (data not shown). Therefore, we decided to use the lower concentration of PMB (30 μg/mL). We also tested the viability of cells in cultures with Polymyxin B using trypan blue stain and we observed that, independent of the presence of PMB, the viability of the cells was about 98 % (data not shown).

**Figure 3:**
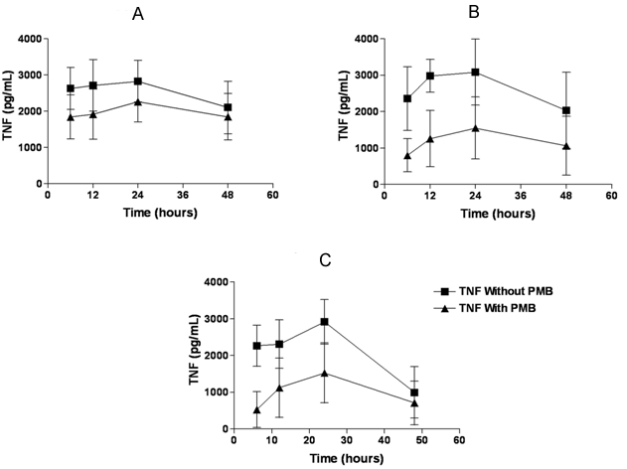
**TNF-α production in PBMC cultures of individuals chronically infected with *S. mansoni *stimulated *in vitro *with *S. mansoni *recombinant proteins in the presence or absence of Polymyxin B**. Figure 3A, B and C show TNF-α production in cultures stimulated with rP24, rSm14 and rSm22.6, respectively. Recombinant proteins were used in the concentration of 10 μg/mL and Polymyxin B at final concentration of 30 μg/mL of culture.

The cytokine production induced by *S. mansoni *recombinant proteins P24, Sm14 and Sm22.6 in the presence or absence of Polymyxin B is shown in Figure 3 (A, B and C, respectively). All recombinant proteins used in this study induced high levels of TNF-α in 6, 12, 24 and 48 h cultures (mean levels higher than 2000 pg/mL), with the exception to Sm22.6 in 48 h culture (mean level 710 ± 1193 pg/mL). With the addition of PMB to the cultures, there was a reduction in the mean levels of TNF-α, mainly when Sm14 and Sm22.6 were used.

**Figure 4:**
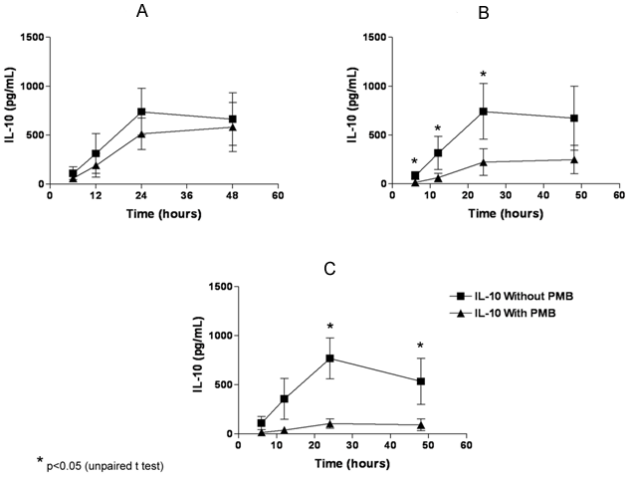
**IL-10 production in PBMC cultures of individuals chronically infected with *S. mansoni *stimulated *in vitro *with *S. mansoni *recombinant proteins in the presence or absence of Polymyxin B**. Figure 4A, B and C show IL-10 production in cultures stimulated with rP24, rSm14 and rSm22.6, respectively. Recombinant proteins were used in the concentration of 10 μg/mL and Polymyxin B at final concentration of 30 μg/mL of culture.

Figure 4A, 4B and 4C show the levels of IL-10 production in cultures stimulated with P24, Sm14 and Sm22.6, respectively. High levels of IL-10 were observed in 24 and 48 h cultures to all three antigens evaluated (above 500 pg/mL). After the addition of PMB, there was higher reduction in IL-l0 production (over 60%) when the cultures were stimulated with Sm14 and Sm22.6 at every time-point of cultures evaluated.

Levels of TNF-α and IL-10 were below the detection limit in unstimulated cultures, and these cytokines were detected in high levels in the supernatants of PBMC cultures stimulated with PHA (data not shown).

## Discussion

Contamination of recombinant proteins cloned in gram-negative bacteria with endotoxin represents a serious problem in laboratory research. In this study, we evaluated a method to neutralize endotoxin as a contaminant of recombinant antigens by reducing production of TNF-α and IL-10 *in vitro*. Polymyxin B is an antibiotic used to prevent septic shock [6,12] and it has been used to neutralize LPS-induced TNF-α production *in vitro *[13]. We used three *S. mansoni *recombinant proteins produced in *E. coli *to test the effect of Polymyxin B on TNF-α and IL-10 production. Before the evaluation of the induction of cytokine production by the recombinant proteins we evaluated the kinetics of TNF-α and IL-10 synthesis in cultures stimulated with LPS, in concentrations similar to what was detected in recombinant proteins. We observed that while TNF-α was produced at high levels in 6 h of cultures and increased after 12 h, IL-10 production started after 12 h of culture and the levels were lower than the levels of TNF-α. IL-10 seems to be produced in response to TNF-α, appearing after 12 h of culture probably to modulate TNF-α production. IL-10 is an anti-inflammatory cytokine, which can modulate TNF-α production and it has been shown that IL-10 down-regulates TNF-α production in models of septic shock [12,14].

The use of Polymyxin B in cultures stimulated with LPS completely abrogated TNF-α production. To a less extent, PMB also down-modulated the TNF-α and IL-10 production when *S. mansoni *recombinant proteins were used. Therefore, we concluded that these proteins are able to induce these cytokine productions by themselves.

Supporting our results, addition of Polymyxin B to murine macrophage cultures stimulated with recombinant proteins produced in bacteria resulted in down-modulation of TNF-α production [8,9]. However, few studies have used Polymyxin B to neutralize TNF-α production in cultures of human cells stimulated with recombinant proteins, and they have not tested the ability of PMB in blocking LPS-induced IL-10 production.

## Conclusion

Many studies have evaluated the immune response induced by recombinant proteins produced in bacteria, few remark on the potential influence of contamination with endotoxin. Here we demonstrated that proteins expressed in bacteria are able to induce high levels of TNF-α and IL-10 production and we demonstrated that Polymyxin B prevented the LPS-induced cytokine production. These results support the use of Polymyxin B in laboratory research that uses recombinant antigens produced in *E. coli *to evaluate the *in vitro *immune response. As some recombinant proteins have the ability to modulate the immune response, they should be produced in a non-bacterial vector for future use as vaccines and treatments to certain diseases.

## Methods

### Study population

Six individuals (4 male and 2 female) chronically infected with *S. mansoni *living in an endemic area in Bahia, Brazil were recruited. Patients were included in the study according to the following criteria: any age or gender and infected with *S. mansoni*.

### *S. mansoni *proteins

The proteins were provided by the Institute of Biological Science, Department of Biochemistry and Immunology, UFMG, Brazil and they were tested for LPS contamination using a commercially available LAL Chromogenic Kit. The concentration of LPS found in these proteins was lower than 0.25 ng/mL (Table 1).

The *S. mansoni *antigens evaluated in this study were Sm22.6, P24 and Sm14. Sm22.6 is a soluble protein from the tegument, present in all life cycle of the worm with the exception of egg [15]. The native form of the P24 is a fraction of the PIII, an antigen obtained from *Schistosoma mansoni *adult worm antigen (SWAP) [16], and Sm14 is a *Schistosoma mansoni *antigen which belongs to the fatty acid-binding protein family and was produced using the pMal-c2 expression system as previously described [17].

The Ethical Committee of the Climério de Oliveira Hospital/Federal University of Bahia approved the present study, and an informed consent was obtained from all study participants or their legal guardians.

### Cell culture and cytokine measurement

Human peripheral blood mononuclear cells (PBMC) were used to test the ability of PMB to neutralize the effect of LPS itself and as contaminant of three different *S. mansoni *recombinant proteins produced in *E. coli *in inducing cytokine production. PBMC were obtained through the Ficoll-Hypaque gradient and adjusted to a concentration of 3 × 10^6 ^cells/ml in RPMI 1640 containing 10% of normal human serum (AB^+^, heat-inactivated), 100 U/mL penicillin, 100 μg/mL streptomycin, 2 mM L-glutamine, 30 mM HEPES (all from Life technologies GIBCO BRL, Gaithersburg, MD). Cells were cultured *in vitro *in the presence or absence of Polymyxin B (10 μg/mL) and were stimulated with LPS (0.14 ng/mL, which is the mean of contaminant concentration in the cultures stimulated with *S. mansoni *antigens), *S. mansoni *recombinant protein rP24, rSm14, rSm22.6 (10 μg/mL) and with the mitogen phytohemaglutinin (PHA) (2 μg/mL). Unstimulated cells were also cultured as a control. Cultures were incubated at 37°C, 5% CO_2 _for 6, 12, 24 and 48 hours. After incubation, the supernatants were collected and maintained at -20°C, for later measurement of cytokines. Levels of TNF-α and IL-10 in culture supernatants were determined by ELISA sandwich technique, using commercially available kits (R&D Systems), and the results were expressed in picograms per milliliter based on a standard curve.

### Addition of polymyxin B to the cultures

Suspension of peripheral blood mononuclear cells (3 × 10^6 ^cells/mL) were pre-incubated with Polymyxin B (CALBIOCHEM, Germany) in the concentration of 10 and 20 μg/mL for 30 minutes at 37°C, 5% CO_2_. They were then incubated with the different recombinant proteins (10 μg/mL) or LPS (0.14 ng/mL) and the cultures were incubated for 6 to 48 hours as described above. Polymyxin B (10 or 20 μg/mL) was also added each 12 hours, during all culture period.

### Statistical analysis

Wilcoxon matched pairs test were used to compare the levels of TNF-α and IL-10 in supernatants of PBMC cultures with or without Polymyxin B. Statistical significance was established at the 95% confidence interval.

## Abbreviations

CD14 – Macrophage receptor

HPLC – High performance liquid chromatography

IL – Interleukin

LBP – LPS binding protein

LPS – Lipopolysaccharides

PAMPs – Pathogen associated molecular pattern

PBMC – Peripheral blood mononuclear cells

PHA – Phytohemaglutinin

PMB – Polymyxin B

TLR-4 – Toll like receptor-4

TNF-α – Tumor necrosis factor-α

## Competing interests

The author(s) declare that they have no competing interests.

## Authors' contributions

LSC, MIA and RRO participated in the immune assays and selection of *Schistosoma mansoni *patients.

LSC, MIA and SCO drafted the manuscript,

LPG, SCO and AMG provided the *S. mansoni *antigens and measured the LPS concentration in the proteins.
